# The genetics of intellectual disability: advancing technology and gene editing

**DOI:** 10.12688/f1000research.16315.1

**Published:** 2020-01-16

**Authors:** Muhammad Ilyas, Asif Mir, Stephanie Efthymiou, Henry Houlden

**Affiliations:** 1 Department of Biological Sciences, International Islamic University Islamabad, Islamabad, 44000, Pakistan; 2 Department of Neuromuscular Disorders, UCL Institute of Neurology, Queen Square, London, WC1N 3BG, UK

**Keywords:** Intellectual Disability, Neurological Disorders, Gene Editing, Mental Retrdation, NGS, WES, CRISPR/Cas9.

## Abstract

Intellectual disability (ID) is a neurodevelopmental condition affecting 1–3% of the world’s population. Genetic factors play a key role causing the congenital limitations in intellectual functioning and adaptive behavior. The heterogeneity of ID makes it more challenging for genetic and clinical diagnosis, but the advent of large-scale genome sequencing projects in a trio approach has proven very effective. However, many variants are still difficult to interpret. A combined approach of next-generation sequencing and functional, electrophysiological, and bioinformatics analysis has identified new ways to understand the causes of ID and help to interpret novel ID-causing genes. This approach offers new targets for ID therapy and increases the efficiency of ID diagnosis. The most recent functional advancements and new gene editing techniques involving the use of CRISPR–Cas9 allow for targeted editing of DNA in *in vitro* and more effective mammalian and human tissue-derived disease models. The expansion of genomic analysis of ID patients in diverse and ancient populations can reveal rare novel disease-causing genes.

## Introduction

Intellectual disability (ID) occurs in the developmental period before the age of 18 years. ID is a heterogeneous group of disorders characterized by significantly impaired intellectual functioning and deficits in adaptive behaviors ^1^. It affects 1–3% of the world population, is the most common developmental disorder, and represents an important socio-economic problem in healthcare. However, owing to the heterogeneity of ID, its frequency ratio changes worldwide ^2^. Diagnosis of ID is also performed with the identification of clinical phenotype symptoms such as delayed speech, hypotonia, and seizures ^3^. In the past decade, the genetic background of ID was believed to be mostly autosomal dominant ( *de novo* mutations) in the outbred countries such as the USA and those in Western Europe, while in the middle-east countries where inbreeding is common, autosomal recessive ID has some preponderance ^4, 5^.

**Figure 1.**
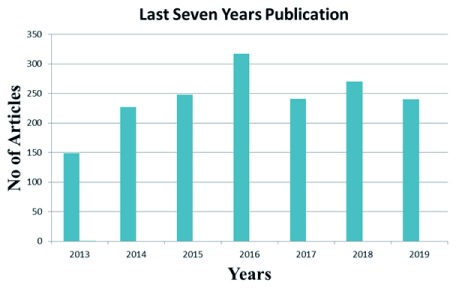
The number of articles on intellectual disability published in the last seven years identified using the PubMed search terms “ID”, “mental retardation”, “next-generation sequencing”, and “exome sequencing”.

Next-generation sequencing (NGS) provided tremendous power to sequence personal genomes and detect a large number of genetic variants. The discovery of disease-causing variants by whole exome or genome sequencing in patients has dramatically changed our perspective on precision medicine ^6^. Through NGS methods, now it is possible to find pathogenic mutations, including novel mutations, associated with ID ^7^. A combined NGS and bioinformatics approach is used to identify novel ID genes and screen candidate ID genes as well. NGS has efficiently expedited ID research and provided new strategies at the clinical level in the last few years, as shown in Figure 1. Genomics England’s PanelApp database ( https://panelapp.genomicsengland.co.uk/panels/285/) shows that around 1,396 genes cause ID (see Table 1, *Extended data*).

## Etiological classification of intellectual disability

**Figure 2.**
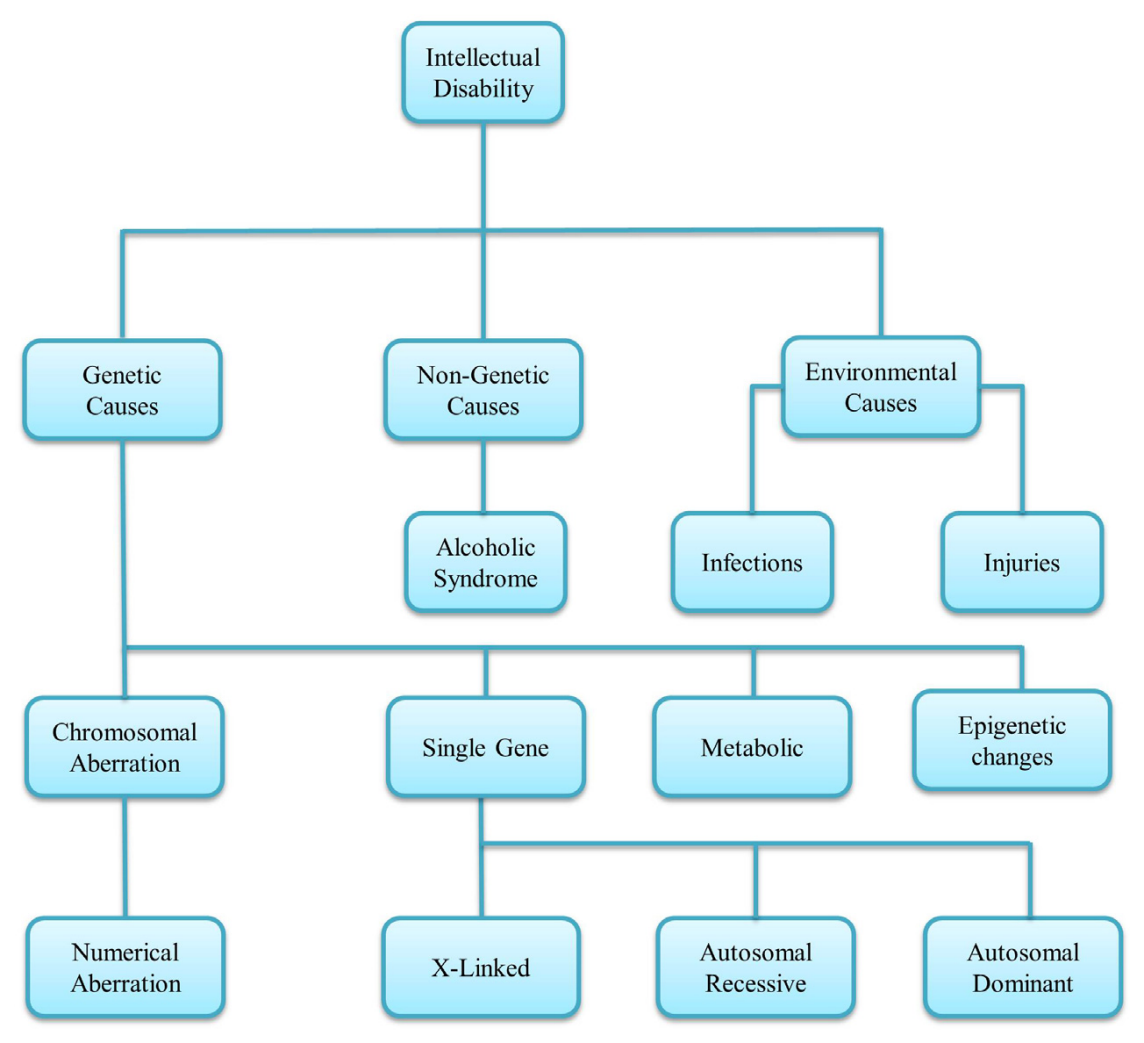
Intellectual disability classification. Multiple factors are involved in intellectual disability including genetic inheritance and environmental conditions.

Multiple factors are involved in causing ID. Genetic factors include genetic variations such as aneuploidies, copy number variations (CNVs), and tandem repeats in specific genes ^8^. DNA is liable to mutation, mediating genetic plasticity. The expansion of tandem repeats can cause a range of disorders associated with ID such as X-linked ID (XLID) ^9^, various ataxias, motor neuron disease, and epilepsy. Emerging data suggest that tandem repeat polymorphisms (TRPs) can contribute to the missing heritability of polygenic disorders ^10– 13^. Furthermore, various metabolic factors, repeat expansions of nucleotides, and mitochondrial DNA variants can also contribute to ID ^7^. Environmental factors such as hazardous chemical exposures, infections during pregnancy, and UV radiation are also reported to cause ID. In addition to these, lack of nutrition, cultural deprivation, childhood diseases such as measles, meningitis, and severe head injury can cause malfunction of the nervous system, leading to ID ^14^. Even though the etiological factors of ID are very broad, as shown in Figure 2, in 50% of individuals the cause of ID is still unknown, but the most prominent causes are genetic ^15^.

### Cytogenetic abnormalities

The identification of genetic factors causing ID has advanced in terms of number and type with recent developments in cytogenetic techniques. The first genetic test used in the investigation of ID was karyotyping ^16^ to identify aneuploidies, such as Down’s syndrome (trisomy 21) and Edwards syndrome (trisomy 18), and large structural re-arrangements such as insertions, deletions, and duplications ^17, 18^. Karyotyping detects only large deletions or insertions owing to its low resolution. Fluorescence *in situ* hybridization (FISH) is used to detect structural abnormalities and numerical changes on chromosomes. Specific probes are used in FISH for the analysis of chromosomal aberration ^19^. A number of studies on ID describe the growing benefits of using NGS in clinics for diagnostic purposes ^20^.

### Chromosomal aberrations

An extra copy of chromosome 21 as a result of an error in cell division can cause a trisomy known as Down’s syndrome ^21^. It is the most frequent form of ID. The clinical features of Down’s syndrome include dysmorphic features, seizures, psychomotor slowing, and congenital malformation. These conditions may not be present in each affected individual ^22^. Edwards syndrome is a trisomy of chromosome 18 characterized by psychomotor and cognitive brain impairment, malformation, and growth deficiency in infants ^23^. The occurrence of Edwards syndrome is 1 out of 8,000 live births. The mortality rate is very high with this condition; only 5–10% of affected children survive after the first year of life ^24^. Patau syndrome is a trisomy of chromosome 13, characterized by malformations of the central nervous system (CNS). The occurrence of this syndrome is 1 in 12,000 in the general population ^23^. The survival rate is very low in infants with trisomy 13, but it depends on the severity of the condition in infants, i.e. whether or not there are cerebral, cardiac, or other congenital malformations ^25^.

### Copy number variation

CNVs are small segments of DNA that vary in number. Usually each individual carries two copies: one that comes from the maternal side and one from the paternal side. Variations in copy number occur through duplications and deletions of the small DNA segments. But not all copy numbers, that are either a deletion or duplication, are pathogenic to humans ^26, 27^. The largest database for CNVs (DGV) is available online ( www.dgv.tcag.ca) ^28^. Decipher is another database used by clinicians to compare clinical and genetic information for the identification and interpretation of pathogenic variants (sequence variants and copy number variants) in patients with ID ^29^. CNVs causing *de novo* and inherited mutations have been associated with ID. A study on a large cohort reported 118 rare *de novo* CNVs ^30^. Analysis of these reported CNVs and candidate genes pinpointed 10 genes with loss of function associated with ID ^31^. CNVs cannot be visibly detected with a light microscope. Array comparative genomic hybridization (CGH) can perform rapid genome-wide analysis at a high resolution and detect CNVs, gain or loss, at the chromosomal level ^32^. Array CGH analysis of unresolved ID cases increased the identification of pathogenic CNVs up to 13% in recent years. Phenotypes associated with these 13% of cases are congenital defects, primary microcephaly, and short stature ^33^. A disorder (Online Mendelian Inheritance in Man [OMIM] #612001) with microdeletion on 15q13.3 shows an ID phenotype with a complex variety of seizures, autism, and psychiatric conditions ^34, 35^. SNP array is also a new technique as compared to array CGH which can detect CNVs ^36^. A novel genetic disorder related to ID with a 3q29 microdeletion was reported using microarray techniques ^37^.

### Whole exome and genome sequencing

Whole exome sequencing (WES) is an efficient technology that can increase the diagnostic yield when searching for alleles causing rare Mendelian disorders. Exome analysis examines the protein’s encoding region, where an estimated 85% of disease-causing mutations are believed to occur ^38^. It has been an invaluable tool in gene discovery for ID. WES performed on three members of a family with autosomal dominant ID (MRD44; 617061) showed a heterozygous mutation, a 1 bp deletion (c.4466delA, NM_007118) in exon 30 of the *TRIO* gene. This mutation resulted in a framshift and premature termination (Gln1489ArgfsTer11) in the *GEFD1* domain. A 9-year-old girl with autosomal dominant ID was also found to carry a *de novo* heterozygous c.3239A-T transversion (c.3239A-T, NM_007118) in exon 19 of the *TRIO* gene, which causes changes in the spectrin repeat domain ^39^. Whole genome sequencing (WGS) allows examination of single-nucleotide variants (SNVs), indels, structural variants (SVs), and CNVs in both the ~1% part of the genome that encodes protein sequences and the ~99% of remaining non-coding sequences. Therefore, WGS has more reliable sequence coverage with more uniformity. It is likely to reveal many novel variants and genes and derive new scientific and clinical findings for ID. With the rapid drop in sequencing cost and the ability of WGS to rapidly produce large volumes of data, it is becoming a powerful tool for genomic research.

## Inherited mutations causing intellectual disability

Single gene disorders are grouped into different types on the basis of inheritance pattern ^40^.

### Autosomal recessive intellectual disability

Autosomal recessive ID is a genetically heterogeneous group of disorders ^41^. Autosomal recessive ID occurs in syndromic and non-syndromic forms. The syndromic type of ID is characterized by intellectual problems occurring with a group of other phenotypic features ^42^. Non-syndromic ID is characterized by a lack of associated pathology. Genes linked with non-syndromic ID are being studied to understand the normal variation in intelligence ^43^. Distinction in intelligence quotient (IQ) is linked with those genes that can also cause large variations in intellectual ability when mutated. Homozygosity mapping is performed to check the autosomal recessive causes of ID in consanguineous families with affected siblings ^44^. OMIM and SysID ( http://sysid.cmbi.umcn.nl/) search results show that 399 genes can cause autosomal recessive ID (see Table 2, *Extended data*).

### Autosomal dominant intellectual disability

The inheritance pattern of autosomal dominant ID is when an individual carries one copy of a mutant allele and one normal allele on a gene. Autosomal dominant ID is caused by heterozygous mutations in different reported genes and CNVs. Tuberous sclerosis, neurofibromatosis, and myotonic dystrophy are autosomal dominant disorders linked with ID ^45^. SNP microarray analysis of the methyl binding domain gene on chromosome 2q23.1 showed that a 200 kb deletion in exon 6 of a female patient was associated with autosomal dominant ID ^46^. It is difficult to find the estimated frequency of mutations in autosomal dominant ID genes. *ARID1B*, *SYNGAP1*, *DYRK1A*, *MED13L*, *KCNQ2*, *CTNNB1*, *STXB1*, *KMT2A*, *PACS1*, *FOXP1*, and *SMARCA2* are the most commonly mutated autosomal dominant ID genes ^47, 48^. Some of these genes are essential for neuronal differentiation in the developing brain and play important roles in synaptic formation and transmission ^49^. The outcomes of our OMIM search show that in total around 180 genes or loci, as reported in the literature, are involved in autosomal dominant ID (see Table 3, *Extended data*).

### X-linked intellectual disability

The human X-chromosome comprises 5% of the human genome, but an increasing number of genetic diseases are associated with the X chromosome; approximately 10–12% of X-chromosome genes have been linked with ID ^50^. X-linked recessive fragile X syndrome occurs on chromosome Xq27.3 in the *FMR1* gene at the 5ʹ untranslated region (UTR) owing to the expansion of CGG trinucleotide repeats ^51^. Fragile X syndrome is clinically characterized as ID; phenotypically, patients show dysmorphic facial features and protruded ears. *FMR1* encodes for a protein that provides RNA stability and plays a key role in brain development and neuronal plasticity. Deficiency of the FMRP protein causes suppression or excitation of GABA that results in low synaptic connections, leading to syndromic features in patients ^52^. The number of new X-linked ID genes identified has increased rapidly over the last few years with the use of NGS. More than 140 known X-linked genes have been reported ^53, 54^.

## Identification of candidate and novel intellectual disability genes

Different techniques have been used to identify novel ID-causing genes over the past few decades. For the identification of novel or candidate genes in affected families, it is necessary to reconstruct the family pedigree and perform some clinical investigation before reaching any conclusion ^55, 56^. The introduction of robust microarray technologies in research has increased the power of identification; SNP arrays can detect small deletions and micro duplications in the probands ^57^. NGS has accelerated the speed of identification of novel ID-causing genes. NGS technology has become very popular over the past 5 years, with a considerable number of new ID genes and candidate genes reported. NGS can detect SNVs and small insertions/deletions in the whole genome.

## *In vitro* and *in vivo* study of intellectual disability

Biological assays can be used to study any undefined variant and its role or pathogenicity. These assays can be used in mutated cells and also directly in patient-derived cells ^58^. To identify the pathogenicity of candidate ID genes, electrophysiological studies of SH-SY5Y neuronal cell lines are a very powerful approach ^59^ to capture early cortical development with high fidelity, thus helping to study genes that are relevant in early cortical development and associated with ID ^60^. *In vivo* studies of candidate ID genes provide additional information regarding pathogenicity. Different animal models can be used to study ID genes. CRBN knockout (CrbnKO) mice have been used to study learning and memory tasks. Loss of CRBN results in memory problems, learning problems when AMPK activity is accelerated, blocking of mTORC1 signaling, and a decreased level of glutamatergic synaptic proteins. These findings show that the CrbnKO mouse is an ideal animal model to understand the molecular mechanisms of learning and memory problems in ID patients ^61^. Zebrafish can also be used as a model organism to study the function of genes associated with ID. Zebrafish have unique features including the rapid development of embryos and easy visualization of the nervous system during developmental stages, which make them an ideal organism to study the function of genes. Overexpression comparison can be performed between the wild-type *PK1A* gene and the zebrafish ortholog of *PK1A*. In this case, zebrafish mutants show severe phenotypes as compared to the wild-type, and a mutant *PK1A* gene has been reported in patients with epilepsy. These results signify that the mutation changes the *in vivo* function of *PK1A* ^62^. In addition, the *TAF1* gene is associated with ID. Functional study of this gene was performed by using a zebrafish knockout model. Severe phenotypes were observed during embryogenesis and neurodevelopment in the zebrafish *TAF1* knockout model ^63^. The heterogeneity of ID makes it very difficult to validate candidate genes as causative ID genes, but *in vitro* and *in vivo* studies provide grounds for conclusively identifying certain genes. Single-cell transcriptomics and quantitative proteomics can also be used to improve our understanding of the global changes in the central nervous system when the genome-edited organism is available.

## CRISPR–Cas9 gene editing tool

**Figure 3.**
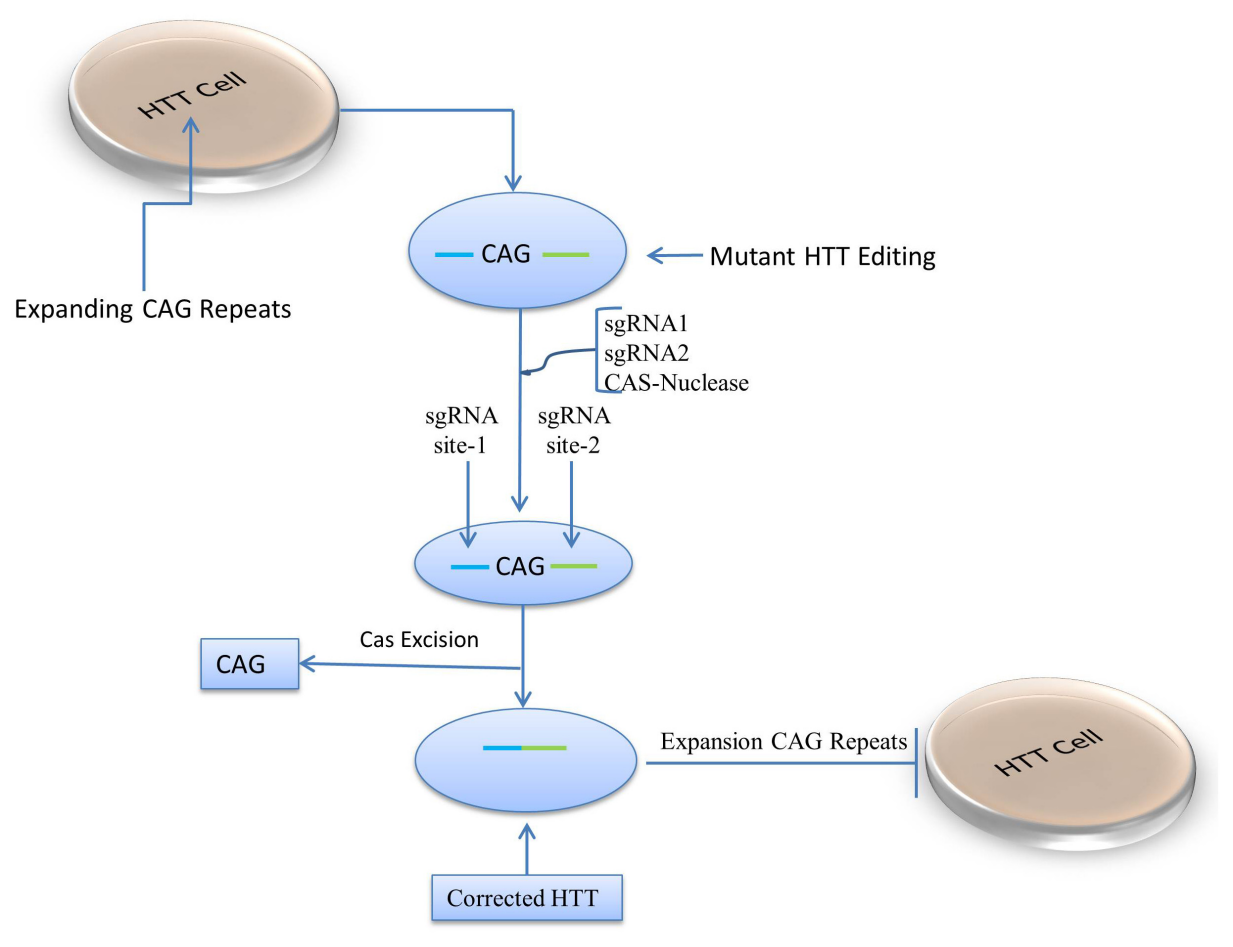
The CRISPR–Cas9 system used to correct extension repeats in the huntingtin gene ( *HTT*) by using single-guide RNA (sgRNA) on both sides of the repeats and Cas9 nuclease, creating nicks and removing the CAG repeats, blocking further extension.

The identification of causal genes and their functional analysis requires further understanding of disease mechanisms, especially for potential therapies, even if we do not have a full understanding of the biological functions. Acknowledging this, clustered regularly interspaced short palindromic repeats (CRISPR)–Cas9 is an RNA control nuclease system that has become essential for gene editing and correcting mutated genes. It provides potential treatment options for genetic disorders that cause ID ( Figure 3). This system has been applied recently to mammalian genomes to stop the expression of the disease gene or to edit mutated genes, thereby correcting the mutation. CRISPR–Cas9 technology will possibly cure diseases that have no treatment option available, such as trinucleotide repeat expansion diseases causing neurological disorders ^64^. CRISPR–Cas9 systems have been used on fragile X syndrome caused by the extension of trinucleotide repeats, which results in deteriorated levels of FMR1 protein and Huntington disease (HD) models. No treatment is available for these conditions ^65^. Induced pluripotent stem cells (iPSCs) of patients with fragile X syndrome in the *FRX* gene upstream of the CGH repeat were targeted with expressed CRISPR–Cas9 nuclease along with single guide RNA (sgRNA) and resulted in the reactivation of the FMR1 protein ^66^. Further studies show that two sgRNA-guided methods that flanked the trinucleotide CGG repeat produced two double-stranded breaks and the recombination of the breaks resulted in the deletion of repeats. The deletion of repeats resulted in an increased FMR1 protein level ^67^. HD is caused by trinucleotide repeat extensions in the coding region of the huntingtin gene ( *HTT*) ^68^. The CRISPR–Cas9 system has been used recently in the treatment of HD. iPSCs derived from a HD patient were corrected by using CRISPR–Cas9 that selectively inactivated the mutant *HTT* gene without altering the normal allele of the same gene ^69, 70^. HD patient fibroblast cell lines were used to show that CAG repeats can be precisely excised using CRISPR–Cas9 nickase from the *HTT* gene ^71^. Strict guidelines must be adopted to avoid the exploitation of the safety and security weakness in genome editing techniques as well as to reduce the risk of off-site editing of genome and epigenetic changes with the help of further research. Adopting appropriate biosafety levels for genome editing to stop contamination is important but so is applying rules to cover the biosecurity of gene editing ^72^. Further studies are needed before the application of gene therapy to the treatment of these diseases.

## Conclusions and the future direction of intellectual disability genetics

The prevalence of ID varies from country to country, and it is especially low in developed countries as compared to less-developed countries ^73, 74^. The identification of genes causing ID rapidly increased over the past 3 to 5 years owing to the use of sophisticated sequencing techniques. The diagnosis of ID patients became easy with new massively parallel sequencing methods and the help of different human variant databases. The heterogeneity of ID makes it difficult for etiological diagnosis; however, WES is likely to be used as the first-line test for ID probands. NGS improves our understanding of the genetic origin of ID. Not all ID-causing genes have been identified yet, but a combined approach of sequencing techniques, functional analysis, and bioinformatics will help to identify new ID-causing genes. This approach will potentially provide a new way of treating ID. Although ID genes can be therapeutically targeted using the CRISPR–Cas-9 system, this method and its application in mammals is still emerging, and many regulatory, methodological, and off-target effects still need to be understood.

The genomic understanding of ID has primarily come from developed countries, and our knowledge in developing countries is very limited. This is important, as many of these countries have a young population and culturally prefer large families, indicating that the burden of ID and other childhood disorders will increase before technology is developed enough to treat these conditions. Therefore, early diagnosis, education, and genetic counseling will be important for families suffering with these conditions. In addition, many of the genes in developing countries are likely to have founder effects that may be amenable to diagnosis in cultural groups and demographic areas, accelerating diagnosis and revealing carrier status to help family planning.

## Abbreviations

CGH, comparative genomic hybridization; CNV, copy number variation; CRISPR, clustered regularly interspaced short palindromic repeats; FISH, fluorescence *in situ* hybridization; HD, Huntington's disease; ID, intellectual disability; iPSC, induced pluripotent stem cell; NGS, next-generation sequencing; OMIM, Online Mendelian Inheritance in Man; sgRNA, single guide RNA; SNV, single nucleotide variation; WES, whole exome sequencing.

## Data availability

### Extended data

Harvard Dataverse: Replication Data for: The Genetics of Intellectual Disability Review Tables, https://doi.org/10.7910/DVN/MMUNLR ^75^

This project contains the following extended data:

- Table 1: Estimated total number of genes involved in Intellectual Disability Genes from Literature search and from genome England Database

Harvard Dataverse: The Genetics of Intellectual Disability, https://doi.org/10.7910/DVN/AEKQOL ^76^

This project contains the following extended data:

- Table 2: List of Genes causing Autosomal Recessive Intellectual Disability

Harvard Dataverse: Replication Data for: The Genetics of Intellectual Disability, https://doi.org/10.7910/DVN/9BJDI6 ^77^

This project contains the following extended data:

- Table 3: List of Autosomal Dominant Genes in Omim and PubMed DataBase

Data are available under the terms of the Creative Commons Zero "No rights reserved" data waiver (CC0 1.0 Public domain dedication).
